# Book Notice

**Published:** 1862-06

**Authors:** 


### BOOK NOTICE.

A MANUAL OF METALLURGY, *more particularly of the precious metals, but including such others as are employed in dental practice.* By GEO. HOGARTH MAKINS, M. R. C. S., F. C. S.; one of the Assayers to the Bank of England, Assayer to the Anglo-Mexican Mints, and Lecturer upon Metallurgy at the Dental Hospital in London, 1862.

This work, as a manual upon the metals, is certainly the best we have ever seen. It describes clearly, yet very briefly, the obvious properties of all the metals, as elements, and then gives the character of many of their combinations with one another and with the non-metallic elements. The treatment of the metals, both analytically and synthetically, we have not anywhere seen so clearly described.

One beauty of this work is, that the ideas are not smothered beneath a mass of useless verbiage, that has to be torn away, before we can get a peep at even the shape of the ideas.

This work was written with special reference to the wants of the dental profession, and well does it fill the place. It should be in the hands of every dentist, no one can afford to be without it, no other work will supply its place. It will be furnished by Robt. Clarke & Co., extensive foreign book dealers of this city. T.

